# The efficacy and cerebral mechanism of intradermal acupuncture for major depressive disorder: a multicenter randomized controlled trial

**DOI:** 10.1038/s41386-024-02036-5

**Published:** 2024-12-08

**Authors:** Xiaoting Wu, Mingqi Tu, Zelin Yu, Zhijian Cao, Siying Qu, Nisang Chen, Junyan Jin, Sangsang Xiong, Jiajia Yang, Shuangyi Pei, Maosheng Xu, Jia Wang, Yan Shi, Lishu Gao, Jian Xie, Xinwei Li, Jianqiao Fang, Xiaomei Shao

**Affiliations:** 1 Department of Acupuncture, The Third Clinical Medical College, The Third Affiliated Hospital of Zhejiang Chinese Medical University, Hangzhou, China; 2 Department of Rehabilitation Medicine, The Second Affiliated Hospital of Zhejiang University, Hangzhou, China; 3 Department of Acupuncture, The Affiliated Hangzhou First People’s Hospital, Hangzhou, China; 4 Department of Radiology, The First Affiliated Hospital of Zhejiang Chinese Medical University, Hangzhou, China; 5 Department of Psychiatry, The Third Affiliated Hospital of Zhejiang Chinese Medical University, Hangzhou, China; 6 Department of Psychiatry, The First Affiliated Hospital of Zhejiang Chinese Medical University, Hangzhou, China; 7 Department of Psychiatry, The Affiliated Hangzhou First People’s Hospital, Hangzhou, China; 8 Department of Acupuncture, Tongde Hospital of Zhejiang Province, Hangzhou, China

**Keywords:** Depression, Outcomes research

## Abstract

New combinations or alternative therapies for major depressive disorder (MDD) are necessary. Intradermal acupuncture (IA) shows promise but requires further investigation regarding its efficacy, safety, and mechanisms. Conducted across 3 centers from November 2022 to January 2024, our randomized controlled trial included 120 participants with moderate to severe MDD, divided into the selective serotonin reuptake inhibitors (SSRIs), SSRIs plus sham IA (SSRIs + SIA), and SSRIs plus active IA (SSRIs + AIA) groups. Acupuncture groups received 10 sessions over 6 weeks at Shenmen (HT7), Neiguan (PC6), Sanyinjiao (SP6) and Taichong (LR3) bilaterally, followed by a 4-week follow-up. The primary outcome was changes in Hamilton Depression Rating Scale-17 (HAMD-17) scores at week 6. Furthermore, healthy controls (HCs) and MDD patients underwent magnetic resonance imaging (MRI) scans for functional connectivity (FC) analysis. After 6 weeks of treatment, the SSRIs + AIA group showed a greater reduction in HAMD-17 score than the SSRIs + SIA group (MD, −4.9 [CI, −7.6 to −2.2], *P* < 0.001) and SSRIs group (MD, −5.1 [CI, −7.8 to −2.3], *P* < 0.001). No serious adverse events occurred. SSRIs + AIA resulted in lower incidences of palpitations (vs.SSRIs + SIA: OR, 0.1% [CI, 0.0–1.0%]; vs. SSRIs: OR, 0.1% [CI, 0.0–0.7%]; *P* < 0.05), somnolence (vs.SSRIs + SIA: OR, 0.1% [CI, 0.0–0.9%]; vs.SSRIs: OR, 0.1% [CI, 0.0–0.7%]; *P* < 0.05), and nausea (vs.SSRIs + SIA: OR, 0.1% [CI, 0.0–1.0%]; vs. SSRIs: OR, 0.1% [CI, 0.0–0.9%]; *P* < 0.05). MDD patients showed abnormal FCs, and IA enhanced FCs between striatum and frontal_inf_tri, and striatum and cerebellum in the MRI study. Overall, IA as adjunctive therapy provides clinical efficacy and safety for MDD, and it may exert antidepressant effects by modulating striatal FCs.

## Introduction

Major depressive disorder (MDD) is characterized by persistent sadness and cognitive impairment, severely impacting daily functioning and potentially leading to self-injury and suicide [1, 2]. Selective serotonin reuptake inhibitors (SSRIs) exert antidepressant effects by promoting neuroplasticity or increasing 5-hydroxytryptamine (5-HT) levels and are recommended as first-line treatments for MDD across all age groups [3–5]. However, more than two-thirds of patients with MDD fail to respond after the initial antidepressants use [6]. It is necessary to adjust treatment regimens in combination with new therapies to address delayed onset, insufficient response, persistent functional impairments, drug resistance, withdrawal syndromes, relapses, and side effects associated with SSRIs [7–9].

Acupuncture is one such alternative or complementary therapy available. Clinical trials have reported significant effects of acupuncture in reducing MDD severity, alleviating somatic symptoms and improving sleep quality [10–12]. Compared to manual or electroacupuncture, intradermal acupuncture (IA) offers advantages such as ease of operation, sustained stimulation, and minimal disruption to daily activities [13]. However, only a limited number of studies have reported the positive effects of IA on MDD [14, 15], and high-quality clinical trials on IA are lacking.

In addition, the antidepressant mechanism of IA for MDD remains unclear. While several theories on the pathogenesis of MDD exist, the most widely accepted involves 5-HT imbalance, which posits that reduced 5-HT levels increase susceptibility to MDD [1, 16]. Seed-based functional connectivity (FC) is one of the prevalent methods for analyzing functional magnetic resonance imaging (fMRI) data, and is often used to characterize the synchronicity between specific brain regions and others [17, 18]. The dorsal raphe nucleus (DRN) and median raphe nucleus (MRN), key brain regions responsible for 5-HT production, are linked to the neurophysiological mechanisms underlying MDD, with FC alterations between these and subcortical regions playing a significant role [19]. Recent studies suggest that the MRN and DRN mediate the anxiolytic and antidepressant effects of acupuncture. For instance, Yang et al. demonstrated that acupuncture improves anxiety-like behaviors by modulating serotonin transporter activity in the DRN [20]. Saiyin et al. found that acupuncture was able to improve depressive-like behavior by regulating BDNF mRNA expression in MRN [21]. Additionally, previous study by our team have shown that 5-HT in the DRN is involved in mediating the analgesic and antidepressant effects of electro-acupuncture [22].

Another prominent theory involves the dysfunction of dopamine reward circuits. Dopaminergic pathways, particularly those involving the ventral tegmental area (VTA) and striatum, are thought to contribute to the abnormal affective states and behaviors observed in MDD [8, 23, 24]. MRI studies have shown structural and functional abnormalities in the striatum are closely linked to MDD onset and severity [25, 26]. Wang et al. have reported changes in striatum-based FC in MDD patients following acupuncture, suggesting that acupuncture exerts antidepressant effects by modulating corticostriatal reward/motivation circuitry [27]. Furthermore, evidence from animal study indicates that acupuncture positively influences anxiety- and depression-like behaviors by regulating neural adaptations within reward circuits in atopic dermatitis mice [28]. In summary, while the antidepressant effects of acupuncture appear to involve both the 5-HT and dopamine systems, there remains a lack of neuroimaging evidence that specifically links IA to specific alterations in brain regions associated with these neurotransmitter systems.

Therefore, we designed a multicenter, prospective, randomized controlled trial (RCT) to observe the clinical efficacy and safety of IA intervention for MDD, and subsequently explored the cerebral mechanism underlying the antidepressant effects of IA using FC analysis of fMRI, focusing on the striatum, VTA, MRN and DRN as region of interests (ROIs).

## Materials and methods

### Study design

This RCT was performed in 3 clinical centers from November 2022 to January 2024, and we followed the Consolidated Standards of Reporting Trials (CONSORT) reporting guideline. This study was reviewed and approved by the Medical Ethics Committee of the Third Affiliated Hospital of Zhejiang Chinese Medical University (approval number: ZSLL-KY-2022-001-01-01), and study protocol has been published [29]. All participants provided written informed consent. This study was registered in and the ClinicalTrials.gov (Identifier: NCT05720637).

### Participants

Participants were recruited, screened and diagnosed by uniformly trained professional psychiatrists according to the International Classification of Diseases 10th Edition (ICD-10) [30]. Inclusion criteria were participants with moderate to severe MDD moderate (score of 17–23) to severe MDD (score≥24) based on the Hamilton Depression Rating Scale-17 (HAMD-17) score [31], between the ages of 18 and 60 years, and administration of SSRIs at least 6 week. Exclusion criteria includes non-monophasic depression, drug or alcohol addiction, severe internal conditions and tumors, positive suicidal tendency, and previously treated with IA or participating in other clinical trials. Healthy controls (HCs) with HAMD-17 < 7 and no history of any psychotic disorders were included. All participants who received MRI scans were right-handed and free of contraindications to MRI. More details are shown in eTable 1.

### Randomization and blinding

One hundred and twenty patients with MDD were randomly allocated by SPSS 25 (SPSS Inc., Chicago, IL, USA) software in a 1:1:1 ratio into 3 groups: only SSRIs, SSRIs plus sham IA (SSRIs + SIA) and SSRIs plus active IA (SSRIs + AIA). Randomization was stratified by gender and age with a block size of 6. An independent assistant then placed the random numbers in sealed envelopes and provided them to participants after the baseline assessment. Participants, outcome assessors and statisticians were blinded to the group allocation, while acupuncturists were not. As for the MRI study, randomization and blinding were not applicable. 20 patients with MDD and 20 gender- and age-match HCs were enrolled [32, 33].

### Procedures

Instructions for SSRIs use were the same in all 3 groups (20 mg/d for fluoxetine, paroxetine and citalopram, 10 mg/d for escitalopram, 50 mg/d for sertraline and 100 mg/d for fluvoxamine). To ensure stable antidepressant treatment, participants have been on SSRIs for at least 6 weeks prior to inclusion in this trial. For those not previously receiving SSRIs, the dose was adjusted to the recommended level. If poor tolerance to the initial SSRI occurred, the psychiatrist adjusted the prescription according to the medication protocol, switching to an alternative SSRI before inclusion. The participant’s antidepressant regimen was stabilized throughout the trial.

Patients in the SIA and AIA groups received acupuncture treatments for approximately 6 weeks, once every 72 h and then removed with 1 day of break, for a total of 10 times. The SIA and AIA were produced by the same company (SEIRIN Co., Japan) with same appearance, but the SIA has a thin silicone pad in the center instead of a needle body. Acupoints Shenmen (HT7), Neiguan (PC6), Sanyinjiao (SP6) and Taichong (LR3) were selected bilaterally according to our previous research [19, 34], and the locations of the acupoints were shown in eTable 2. Patients in the AIA group were instructed to press each acupoint 3–4 times a day for 1 min at 4-h intervals, with as much stimulation as could be tolerated, while patients in the SIA group were not pressed. What’s more, patients did not receive any other antidepressants beyond SSRIs, but temporary administration of sedative-hypnotics was allowed if necessary, and all patients were instructed to keep medication diaries.

### Clinical outcomes

The primary outcome was defined as a reduction in the HAMD-17 score at the 6th week relative to baseline.

The secondary outcomes were changes in scores from baseline to each measurement point (at the 3rd, 6th and 10th week) on the Self-Rating Depression Scale (SDS) and Pittsburgh sleep quality index (PSQI) [35–37]. Changes from baseline in HAMD-17 total and five factor scores (anxiety/somatization, cognitive impairment, retardation, sleep disturbance and weight) [38] at the 3rd and 10th week were also reported. Additional outcomes were IA compliance rate ([actual treatment times/total treatment times] × 100%, 6th week), IA blind success rate (6th week) and adverse events (AEs) rate (10th week). Details of all AEs in this study were recorded on the case report file (CRF) throughout the treatment and follow-up periods, and patients were managed properly and timely. Besides, acupuncture expectancy (5-point method) was assessed at baseline, whereas acupuncture compliance (compliance rate = (actual treatment times/total treatment times) × 100%) and blinding were assessed at the end of treatment.

### fMRI procedures and data acquisition

The non-repeated event-related (NRER) paradigm was applied in this MRI study [39, 40]. HCs underwent only a 8 min rs-fMRI scan, while patients with MDD underwent 8 min rs-fMRI scans respectively before and after the first AIA stimulation. Acupoints were selected consistent with the trial, and the order of stimulation was PC6, HT7, SP6, and LR3, from right to left (all taken bilaterally), with a stimulation time of 1 min for each acupoint and a stimulation frequency of 60 times per minute. Every MDD patient received a total of 8 minutes of continuous AIA stimulation before undergoing the second rs-fMRI scan. All AIA stimulation was performed by two standardized trained acupuncturists, and all MRI scans were operated by the same professional radiologist, and participants were given uniform instructions: lie flat and relaxed, keep eyes closed but stay awake. The experimental paradigm was shown in eFig. 1.

Images were obtained by a 3.0 Tesla MR scanner (GE Discovery MR750, GE Healthcare, Chicago, IL, United States). Structural images were acquired by 3D T1BRAVO sequence: time of repetition (TR) = 8.2 ms, time of echo (TE) = 3.2 ms, flip angle = 12, field of view (FOV) = 256 mm × 256 mm, matrix = 512 × 512, and slice thickness = 1 mm. Resting state functional data was obtained via echo-planar T2*-weighted image (EPI) for 240 time points in the sequence: TR = 2000 ms, TE = 35 ms, flip angle = 90, slice thickness = 5 mm, slice gap = 1 mm, FOV = 256 mm × 256 mm, and matrix = 64 × 64.

To investigate the role of the dopamine reward pathway and the 5-HT system in mediating the antidepressant effects of IA, we chose the striatum, VTA, DRN and MRN as region of ROIs for the FC analysis. The striatum is subdivided into six subregions: inferior ventral caudate/nucleus accumbens (VSi), superior ventral caudate (VSs), dorsal caudate (DC), dorsal caudal putamen (DCP), dorsal rostral putamen (DRP), and ventral rostral putamen (VRP). All ROIs were defined based on previous studies [41–43] as shown in eTable 3.

### Clinical data analysis

On the basis of our preliminary pre-experiment, it was predicted that the mean reduction in HAMD-17 for each group at the end of the intervention would be 12.1, 9.7, and 9.5 with standard deviations of 2.7, 3.7, and 3.2, respectively. When *α* = 0.05, 1-β = 0.9 and the sample sizes of the 3 groups were equal, a sample size of 32 per group was calculated using the PASS 15 software (NCSS LLC., Kaysville, UT, USA). Estimating a dropout rate of 20%, a total of 120 patients (40 patients per group) were enrolled in the study. Missing data from scale assessments were filled in using the last observation carried forward (LOCF).

Normally distributed continuous variables were presented as means and SDs, and binary or ordinal variables were described as numbers and percentages. Differences between the 3 groups were evaluated by the one factorial *ANOVA* test, repeatedly measured data were assessed by the two-way repeated measures *ANOVA* test, the *Bonferroni* test and mean difference (MD) with 95% confidence intervals (95% CIs) were used in pair-wise comparisons between any two groups. Effect sizes were calculated using *Cohen’s d*, with 0.2, 0.5, and 0.8 considered small, moderate, and large, respectively. The Chi-square (*χ*^2^) test and odds ratio (OR) with 95% CIs were used for the categorical variables. All analyses were carried out by using the SPSS 25.0 software (IBM Corporation, Armonk, NY, USA), and statistical significance was defined as a value of *P* < 0.05.

### FC analysis

MRI data were preprocessed using statistical parametricplotting software (SPM12) and the DPARSF toolbox (both implemented by MATLAB R2016b, Math Works, Inc., Natick, MA, USA). The default preprocessing pipeline was used for seed-to-voxel FC analysis. The frist 10 volumes were discarded for stabilization of the initial signal, then slice timing and head motion correction were performed for the remaining volumes. Data from participant with head movement greater than 2.0 mm translation or 2.0° rotation were removed in futher analysis. Then, the data were normalized to the Montreal Neurological Institute (MNI) template, smoothed with a 6-mm Gaussian kernel, and processed by regression, detrending and band-pass filtering (0.01-0.08 Hz). Two-sample *T*-test was applied to examine the ROI seed-based FC differences between MDD patients and HCs. Paired *T*-test was used to detect the regulation patterns of the ROI in MDD patients before and after AIA intervention. The contrast map was corrected using Gaussian random field (GRF, with voxel *P* < 0.05, cluster *P* < 0.05).

## Results

### Baseline characteristics

**Fig. 1:**
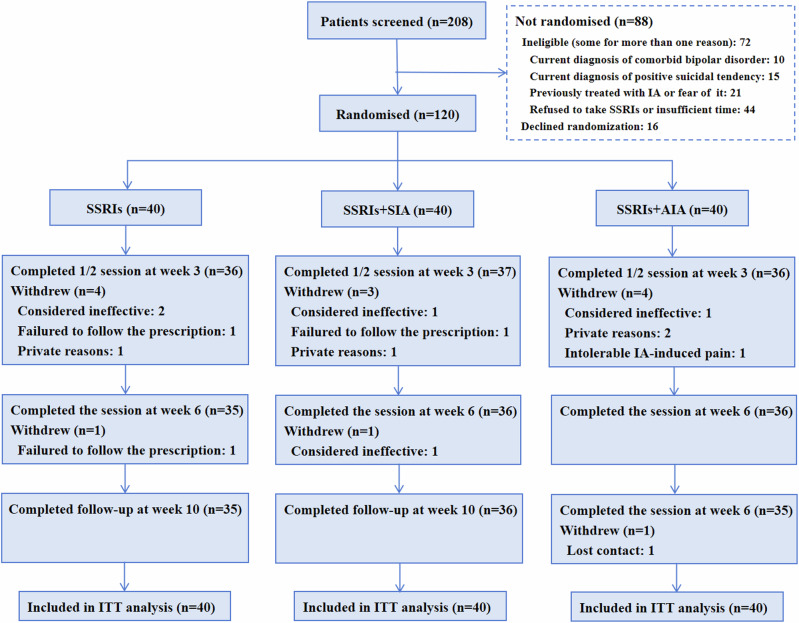
Trial flow chart. SSRIs indicates selective serotonin reuptake inhibitors, SIA indicates sham intradermal acupuncture, AIA indicates active intradermal acupuncture and ITT indicates intention-to-treat.

In this RCT, a total of 208 MDD participants were screened, 120 (57.7%) eligible participants were randomly assigned and included in the intention-to-treat (ITT) analysis, 109 (90.8%) completed half of the session and were included in the modified ITT (mITT) analysis, and 106 (88.3%) completed the session and follow-up assessment. Fourteen MDD participants (5 in the SSRIs group, 4 in the SSRIs + SIA group, and 5 in the SSRIs + AIA group) dropped out of the trial, and the dropout rates in the 3 groups were not statistically different (shown in Fig.1). Anxiety disorders accounted for the highest proportion of MDD comorbidities at 68.3%, followed by sleep disorders at 57.5% (shown in eTable 4). The mean age of the study population was 32.1 years, mean disease duration was 25.1 months, and mean acupucture expectation was 3.0 points. Overall, similar baseline characteristics among the 3 groups are shown in Table 1. In addition, 38 (31.7%) believed that IA would be very effective in MDD symptom improvement, with no significant difference among 3 groups (shown in eTable 5).

**Table 1:** Baseline characteristics in the RCT.

Characteristics	SSRIs (n = 40)	SSRIs + SIA (n = 40)	SSRIs + AIA (n = 40)	All participates (n = 120)
Age (years), mean (SD)	35.2 (13.0)	31.2 (10.8)	30.0 (10.8)	32.1 (11.7)
Sex, no. (%)				
Female	31 (77.5)	32 (80.0)	32 (80.0)	95 (79.2)
Male	9 (22.5)	8 (20.0)	8 (20.0)	25 (20.8)
BMI (kg/m^3^), mean (SD)	21.1 (2.4)	21.8 (3.4)	21.0 (2.7)	21.3 (2.9)
Education, no. (%)				
Primary and secondary	19 (47.5)	16 (40.0)	19 (47.5)	54 (45.0)
Tertiary and above	21 (52.5)	24 (60.0)	21 (52.5)	66 (55.0)
Course of disease (months), mean (SD)	28.7 (43.5)	22.3 (34.5)	24.3 (26.5)	25.1 (35.3)
Use of SSRIs, no. (%)				
Fluvoxamine	15 (37.5)	14 (35.0)	16 (40.0)	45 (37.5)
Fluoxetine	7 (17.5)	10 (25.0)	7 (17.5)	24 (20.0)
Sertraline	8 (20.0)	9 (22.5)	10 (25.0)	27 (22.5)
Escitalopram	9 (22.5)	7 (17.5)	6 (15.0)	22 (18.3)
Paroxetine	1 (2.5)	0 (0.0)	1 (2.5)	2 (1.7)
Acupucture expectation score, mean (SD)	2.8 (1.0)	3.1 (0.9)	3.2 (0.8)	3.0 (0.9)
HAMD-17 score, mean (SD)	21.1 (3.6)	20.1 (3.7)	21.5 (4.4)	20.9 (3.9)
SDS score, mean (SD)	68.3 (7.8)	66.8 (8.0)	72.7 (9.0)	69.2 (8.6)
PSQI score, mean (SD)	15.4 (4.4)	15.3 (3.9)	14.7 (3.5)	15.1 (4.0)

*RCT* randomized controlled trial; SSRIs, selective serotonin reuptake inhibitors, *SIA* sham intradermal acupuncture, *AIA* active intradermal acupuncture, *BMI* body mass index, *HAMD-17* Hamilton Depression Rating Scale-17, *SDS* Zung Self-Rating Depression Scale, *PSQI* Pittsburgh Sleep Quality Index.

The MRI study included 20 patients with MDD and 20 HCs matched for age and gender (shown in eTable 6), of which 17 patients with MDD completed both pre- and post-IA intervention MRI scans, and 20 HCs completed one MRI scan (shown in eFig. 2). In addition, 2 patients with MDD were excluded from the final analysis because of large head motions.

### Clinical outcomes

The mean change in HAMD-17 total score relative to baseline after 6 weeks of treatment was −9.6 (SD, 4.9) for SSRIs + AIA group, −4.8 (SD, 4.6) for SSRIs + SIA group, and −4.6 (SD, 4.7) for SSRIs group. The participants who received SSRIs + AIA had a greater reduction in HAMD-17 total score than that in the SSRIs + SIA group (MD, −4.9 [CI, −7.6 to −2.2]) and SSRIs group (MD, −5.1 [CI, −7.8 to −2.3]) (shown in Table 2).

**Table 2:** Primary and Secondary Outcomes in the Modified Intention-to-Treat Analysis.

Variables	SSRIs (n = 36)	SSRIs + SIA (n = 37)	SSRIs + AIA (n = 36)	Inter-group differences^a^		Repeatedly measured data^b^		Between-group comparisons			
				*F*	*P*	*F*	*P*	SSRIs + AIA vs. SSRIs		SSRIs + AIA vs. SSRIs + SIA	
								MD (95% CI)^c^	Effect size^d^	MD (95% CI)^c^	Effect size^d^
Primary outcome											
HAMD-17 score changes at week 6, mean (SD)	−4.6 (0.8)^†^	−4.8 (0.8)^†^	−9.6 (0.8)^†*#^	13.19	<0.001			−5.1 (−7.8 to −2.3)	−1.04	−4.9 (−7.6 to −2.2)	−1.03
Secondary outcome											
HAMD-17, mean (SD)											
Week 3	−3.3 (0.6)^†^	−2.8 (0.6)^†^	−5.2 (0.6)^†#^	4.70	0.011			−1.9 (−3.9 to 0.1)	−0.53	−2.4 (−4.4 to −0.4)	−0.71
Week 10	−7.2 (0.9)^†^	−6.5 (0.9)^†^	−10.6 (0.9)^†^*^#^	5.84	0.004			−3.4 (−6.5 to −0.3)	−0.65	−4.1 (−7.2 to -1.0)	−0.73
SDS, mean (SD)						8.61	<0.001				
Week 3	−3.6 (7.2)^†^	−4.8 (5.7)^†^	−11.1 (8.3)^†^*^#^	11.28	<0.001			−7.4 (−11.5 to −3.3)	−0.95	−6.2 (−10.3 to -2.2)	-0.88
Week 6	−4.7 (8.4)^†^	−6.2 (8.6)^†^	−17.0 (10.1)^†^*^#^	19.76	<0.001			−12.3 (−17.4 to −7.1)	−1.32	−10.8 (−16.0 to −5.6)	-1.15
Week 10	−8.5 (10.1)^†^	−10.2 (11.1)^†^	−18.5 (11.3)^†^*^#^	8.88	<0.001			−10.1 (−16.3 to −3.8)	−0.96	−8.3 (−14.5 to −2.1)	-0.76
PSQI, mean (SD)						0.74	0.593				
Week 3	−1.6 (3.0)	−1.1 (4.3)	−2.0 (3.2)^†^	0.64	0.529			−0.4 (−2.5 to 1.6)	−0.14	-0.9 (-3.0 to 1.1)	−0.25
Week 6	−2.7 (3.5)^†^	−2.7 (3.5)^†^	−4.1 (3.4)^†^	1.45	0.239			−1.4 (−3.7 to 0.9)	−0.40	−1.4 (−3.7 to 0.9)	−0.33
Week 10	−3.8 (3.4)^†^	−3.5 (4.6)^†^	−4.2 (4.3)^†^	0.32	0.729			−0.4 (-2.8 to 1.9)	−0.12	−0.8 (−3.1 to 1.6)	−0.17

*SSRIs* selective serotonin reuptake inhibitors, *SIA* sham intradermal acupuncture, *AIA* active intradermal acupuncture, *SDS* Zung Self-Rating Depression Scale, *PSQI* Pittsburgh Sleep Quality Index, *MD* mean difference.

*P < 0.05 vs. SSRIs group, ^#^*P* <0.05 vs. SSRIs + SIA group; ^†^*P* < 0.05 for intra-group comparisons.

^a^Inter-group differences were evaluated by the one factorial *ANOVA* test.

^b^Repeatedly measured data (10 weeks’ period) were assessed by the two-way repeated measures ANOVA test.

^c^Between-group comparisons were adjusted by Bonferroni test.

^d^Effect size was calculated using Cohen’s *d*.

Overall, the HAMD-17, SDS and PSQI scores decreased over time. Significant differences across groups were observed in HAMD-17 and SDS scores at the 3rd, 6th, and 10th week (*P* < 0.05) (shown in Table 2). The improvement of the anxiety/somatization and retardation factor scores in the SSRIs + AIA group were significantly better than those of SSRIs + SIA (at the 6th and 10th week) and SSRIs group (at the 6th week) (*P* < 0.05) (shown in eTable 7). Similar to the change in HAMD-17 total score, SSRIs + AIA achieved greater reductions in SDS scores than SSRIs + SIA (MD, −10.8 [CI, −16.0 to −5.6) and SSRIs (MD, −12.3 [CI, −17.4 to −7.1) at the 6th week. The inter-group difference in PSQI was not statistically significant at any time (*P* > 0.05). Intra-group comparisons showed that HAMD-17 and SDS scores were significantly decreased compared to their respective baselines in all 3 groups since the 3rd week (*P* > 0.05). In terms of changes in PSQI scores, significant differences were observed earlier in the SSRIs + AIA group (at the 3rd week) than other groups (both at the 6th week) (shown in Table 2). Throughout the trial, patients were allowed to take comorbid medications temporarily, and the most common sedative-hypnotics were Alprazolam and Oxazepam (shown in eTable 8.) For other outcomes, there were no statistically significant differences in blind evaluation, and IA compliance among the 3 groups (*P* < 0.05) (shown in eTable 9).

No serious adverse events were recorded. Compare to SSRIs + SIA and SSRIs, SSRIs + AIA resulted in a lower frequency of palpitations (vs.SSRIs + SIA: OR, 0.1% [CI, 0.0–1.0%]; vs.SSRIs: OR, 0.1% [CI, 0.0–0.7%]), somnolence (vs.SSRIs + SIA: OR, 0.1% [CI, 0.0–0.9%]; vs.SSRIs: OR, 0.1% [CI, 0.0–0.7%]), and nausea (vs.SSRIs + SIA: OR, 0.1% [CI, 0.0–1.0%]; vs.SSRIs: OR, 0.1% [CI, 0.0–0.9%]) (shown in Table 3).

**Table 3:** Adverse events.

Adverse events, no. (%)	SSRIs (n = 40)	SSRIs + SIA (n = 40)	SSRIs + AIA (n = 40)	SSRIs + AIA vs. SSRIs, OR (95% CI)	SSRIs + AIA vs. SSRIs + SIA, OR (95% CI)
Serious adverse events	0 (0.0)	0 (0.0)	0 (0.0)	_	_
Tiredness	1 (2.5)	1 (2.5)	0 (0.0)	NA	NA
Dizziness	1 (2.5)	2 (5.0)	1 (2.5)	1.0 (0.1–16.6)	0.5 (0.0–5.6)
Headache	2 (5.0)	1 (2.5)	2 (5.0)	1.0 (0.1–7.5)	2.1 (0.2–23.6)
Palpitations	9 (22.5)	7 (17.5)	1 (2.5)*^#^	0.1 (0.0–0.7)	0.1 (0.0–1.0)
Dry mouth	1 (2.5)	0 (0.0)	1 (2.5)	1.0 (0.1–16.6)	NA
Tremor	0 (0.0)	0 (0.0)	1 (0.0)	NA	NA
Sweating	1 (2.5)	1 (2.5)	0 (0.0)	NA	NA
Somnolence	9 (22.5)	8 (20.0)	1 (2.5)*^#^	0.1 (0.0–0.7)	0.1 (0.0–0.9)
Insomnia	7 (17.5)	6 (15.0)	6 (15.0)	0.8 (0.3–2.7)	1.0 (0.3–3.4)
Nausea	8 (15.0)	7 (17.5)	1 (2.5)*^#^	0.1 (0.0–0.9)	0.1 (0.0 to 1.0)
Acupoints pain	0 (0.0)	0 (0.0)	1 (2.5)	NA	NA

*SSRIs* selective serotonin reuptake inhibitors, *SIA* sham intradermal acupuncture, *AIA* active intradermal acupuncture, OR odds ratio.

*P < 0.05 vs. SSRIs group; ^#^*P* <0.05 vs. SSRIs + SIA group.

### rs-FC results

**Fig. 2:**
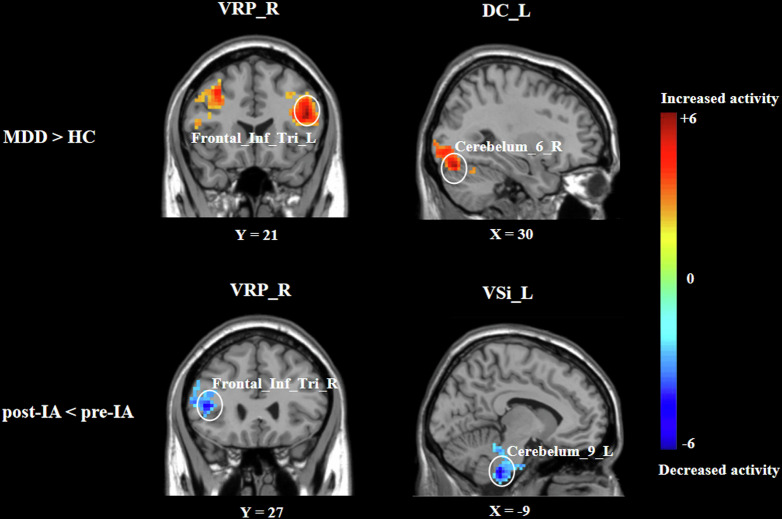
Striatal functional connectivity. MDD indicates major depressive disorder, HC indicates healthy controls, IA indicates intradermal acupuncture, VRP indicates ventral rostral putamen, DC indicates dorsal caudate, VSi indicates inferior ventral caudate/nucleus accumbens, L indicates left, and R indicates right.

Compare with HCs, patients with MDD showed significantly decreased FC between Striatum and left Frontal_Inf_Orb, right_Frontal_Mid_Orb, left Frontal_Sup_Orb, left Frontal_Sup, left Frontal_Sup_Medial, left Precuneus, bilateral Occipital_Mid, right Insula and right Rectus, and increased FC between Striatum and left ParaHippocampal, right Paracentral_Lobule, left Postcentral, left Frontal_Inf_Tri, right Cerebelum_6, left Lingual and bilateral Frontal_Mid. With the VTA as ROI, FC increased between left VTA and left Precuneus, as well as right VTA and left Postcentral. Additionally, FC increased between DRN and left Precentral, also between MRN and right Frontal_Inf_Orb, and decreased FC between right Cerebelum_4_5 (shown in eTable 10, Fig. 2).

Comparison of pre-IA and post-IA results revealed that IA decreased FC between Striatum and left Cerebelum_9, right Frontal_Inf_Tri, as well as between right VTA and right Caudate; IA increased FC between DRN and right Calcarine, and decreased FC between MRN and left Frontal_Sup_Orb (shown in eTable 11, Fig. 2).

## Discussion

Our RCT results showed that AIA had strong effects on MDD symptoms, particularly anxiety/somatization and retardation (as measured by the HAMD-17), outperforming both SIA and SSRIs. The changes in the SDS total score mirrored the trends observed with the HAMD-17. Although all groups exhibited improvements in sleep quality (as measured by the PSQI), no significant differences were observed among the 3 groups at any time point. Importantly, no serious adverse events were reported in any group, and the AIA group experienced markedly fewer adverse events, such as palpitations, fatigue and nausea, compared to other groups. High treatment compliance, exceeding 90%, was observed in the acupuncture groups. These findings indicate that AIA provides therapeutic effects beyond the placebo effect, and this combined therapy showed superior antidepressant efficacy, faster response time, and greater safety compared to SSRIs alone.

Previous systematic reviews have highlighted the positive effects of acupuncture on MDD [11, 12, 44]. An 8-week non-randomized trial reported significant reductions in HAMD-24 total scores, anxiety/somatization and desperation factor scores in an electroacupuncture plus SSRIs group compared to SSRIs alone [45]. Comparable benefits of electroacupuncture on anxiety/somatization, retardation and sleep disturbance have been documented [10]. AIA, a form of superficial acupuncture, shows similar antidepressant effects. Notably, Noda et al. reported AIA improved Beck Depression Inventory scores in MDD patients compared to SIA [14]. Wang et al. also found that SDS total scores, HAMD-17 total scores and sleep factor scores were significantly lower in the AIA group than in the SIA group after a 2-week intervention [15]. Our study aligns with these findings, showing significant improvements in SDS and HAMD-17 scores, particularly in anxiety/somatization and retardation. However, AIA did not significantly improve sleep quality, as measured by both the HAMD-17 sleep factor and PSQI scores. This may be due to differences in acupoint selection,as AIA at auricular acupoints is reported to affect sleep through vagus nerve stimulation [15, 46]. Additionally, the depth of needling in our study differed from that used in manual or electroacupuncture in other studies, which may have influenced the results. The therapeutic benefits of AIA emerged at week 3 and persisted until week 10, indicating a potential prolonged effect, consistent with other studies showing sustained benefits of auricular acupuncture [46].

Most studies have proved that manual or electroacupuncture for MDD accelerates SSRI onset and significantly reduces adverse effects [12, 47]. Our findings provide such evidence for IA intervention for the first time. By week 3, reductions in HAMD-17 and SDS scores in the AIA group exceeded those in the SSRIs group at week 6 (HAMD-17: −5.2 vs. −4.6; SDS: −11.1 vs. −4.7). Although there were no significant difference in PSQI scores among the three groups, the AIA group showed a therapeutic advantage at week 3 compared to the baseline, earlier than the other two groups. Furthermore, the AIA intervention significantly decreased the incidence of palpitations, fatigue and nausea, possibly due to sympathetic and vagus nerves modulation via the median nerve at acupoint PC6 [48]. Only one case (2.5%) in the AIA group experienced intolerable pain at the acupoints, with no adverse events such as dizziness, hematoma, and bleeding, which are common with traditional acupuncture, indicating that AIA is a safer therapy. In summary, our RCT provided strong evidence for the efficacy and safety of AIA for MDD.

Our study explored the potential cerebral mechanisms underlying the antidepressant effects of AIA, focusing on the dopamine reward and 5-HT systems. Dopamine, primarily originating from the midbrain VTA, projects to the prefrontal cortex and the limbic system (nucleus accumbens, NAc; also known as VSi) [49, 50]. The DRN and MRN, rich in 5-HT, also plays a crucial role in regulating emotions, cognition, behavior, and motivation. Hyperactivity in specific brain regions is associated with the onset and progression of MDD, including the left parahippocampal, left hippocampus, left amygdala, and left medial orbital frontal gyrus [51]. Our study found that compared to HC, patients with MDD show increased FCs between the striatum, VTA, DRN and MRN and regions in the frontal, parietal, temporal lobes, or the cerebellum, which are involved in emotional regulation and social cognition. Notably, enhanced FC between the striatum and frontal_inf_tri was attenuated following AIA intervention, suggesting the striatum-prefrontal cortex circuit was modulated by AIA. In addition, the cerebellum, traditionally associated with motor control, is increasingly recognized for its role in cognitive and emotional processing of negative stimuli [52]. Abnormal cerebellar activity is frequently observed in MDD, and cerebellar dysfunction can lead to emotional and behavioral abnormalities [53]. Current theories propose that the cerebellum regulates emotions through cerebellar-cortical or cerebellar-limbic system circuit [54]. Zhang et al. found that hypothalamo-cerebellar-amygdala circuit might alleviate movement-dependent anxiety, underscoring the cerebellar-limbic system’s role in emotion regulation [55]. Consistent with this, we observed that the cerebellar-limbic system (striatum) circuit mediated the antidepressant effects of AIA. Specifically, MDD patients showed enhanced FC between the striatum and cerebellum compared to HCs, which decreased following AIA intervention. Additionally, AIA also modulated FC of VTA, MRN and DRN, though further research is required to confirm their roles in AIA’s antidepressant effects.

Acupuncture has been shown to produce sustained effects, with neural responses persisting for several minutes after needle removal [56]. Price et al. confirmed that the analgesic effects of acupuncture actually peak some time after the needle has been removed [57]. Consequently, research on these sustained effects, particularly using NRER design, has gained prominence [40, 58]. Wei et al. demonstrated that electro-acupuncture induced stronger and broader brain responses in MDD patients 15 minutes after needle removal (sustained effects) than during needling (instant effects) [59]. The sustained effects of acupuncture refer to short-term neural responses occurring within minutes to tens of minutes after needle removal, typically associated with improvements in mood and cognitive function. Long-term effects, on the other hand, involve neuroadaptive changes that develop over extended periods, potentially leading to lasting alterations in brain structure and function. For instance, Wang et al. found that acupuncture modulates FC between the amygdala and anterior cingulate cortex, contributing to long-term improvements in MDD [60]. Despite differences in time scales and physiological mechanisms, both sustained and long-term effects significantly improve MDD symptoms by moderating neural network function and structure. In this study, we adopt an NRER-based design to explore the sustained effects of IA on MDD, laying the groundwork for future research on its long-term effects. Future research will systematically compare the instant, sustained, and long-term effects of IA to clarify its central mechanisms in MDD.

### Limitations

First, the treatment period and follow-up time were relatively short, leaving the long-term antidepressant effect of AIA combination therapy unknown. Second, we did not analyze SSRIs dosage as a variable, potentially limiting the generalizability of our findings. Third, in fMRI studies, factors such as pain or visual disturbances from AIA could influence brain activity, but placebo or SIA were not selected for negative control in this study. Fourth, many MDD patients declined a second MRI scan after the 6-week intervention, necessitating a shift to a NRER design, which may differ from long-term effect studies.

## Conclusions

Combining AIA with SSRIs effectively alleviated depressive symptoms, with favorable treatment adherence and safety. Compared to SSRIs alone, the combination effectively accelerated the onset of SSRI effects and reduced adverse effects. Furthermore, MDD patients exhibited FC abnormalities in brain regions associated with the dopamine reward and the 5-HT system, and the striatum-frontal cortex (frontal_inf_tri) and cerebellar-limbic system (striatum) circuits appear to mediate the antidepressant effects of AIA.

## Supplementary information

Supplement Material

## Data Availability

Due to ethics restrictions, the data supporting the results of this study are not publicly available, but they are available to the applicant upon reasonable request to the corresponding author: shaoxiaomei@zcmu.edu.cn.
