# Supplementary material for: The efficacy and cerebral mechanism of intradermal acupuncture for major depressive disorder: a multicenter randomized controlled trial
Source: Neuropsychopharmacology. 2024 Dec 8;50(7):1075–83. doi: 10.1038/s41386-024-02036-5 (PMC12089605; doi:10.1038/s41386-024-02036-5)
Supplement: Supplementary file 1 — Supplement Material [file 41386_2024_2036_MOESM1_ESM.docx]

****Supplement Figure****

****eFig.1. The Non-repeated Event-related (NRER) Paradigm in the MRI study****


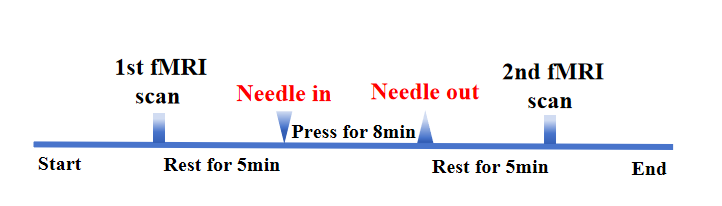


****eFig.2. MRI study flow chart****


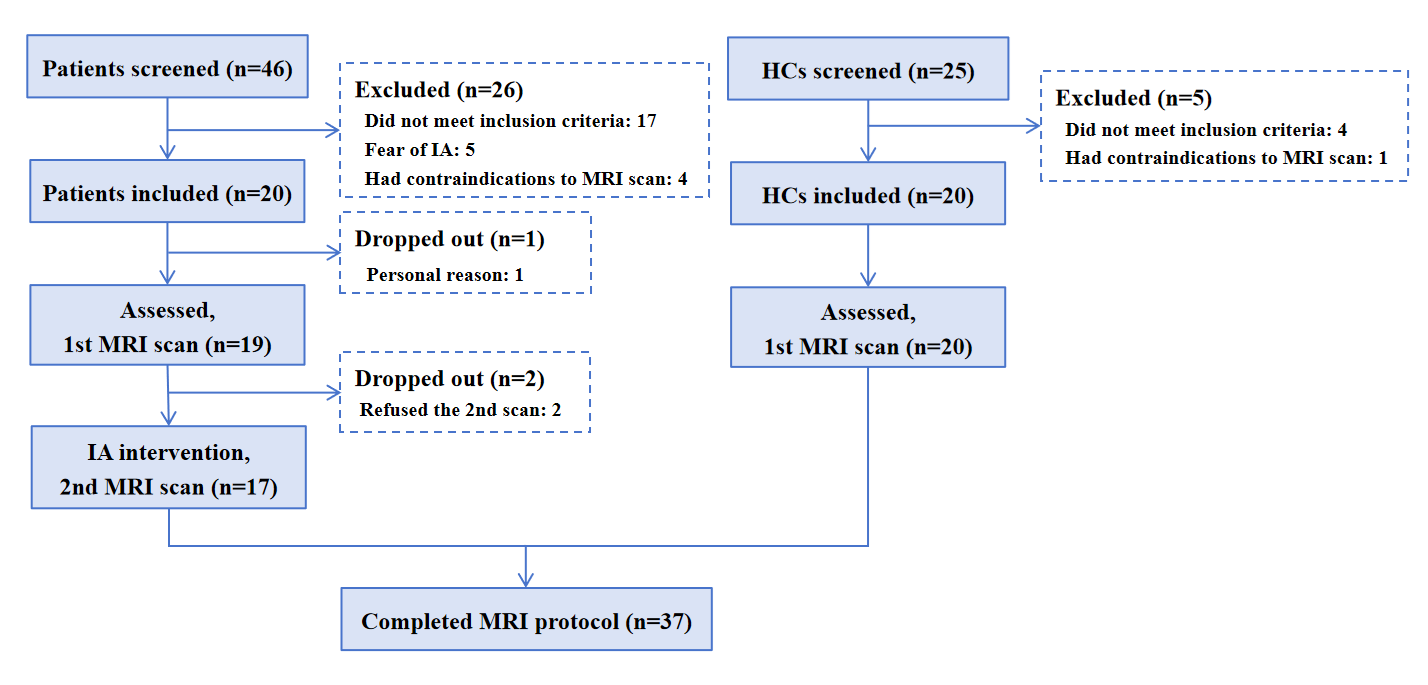


****Supplement Table****

****eTable 1. Inclusion and Exclusion Criteria****

| **Inclusion Criteria** | **Exclusion Criteria** |
| --- | --- |
| - Patients diagnosed with MDD according to the International Classification of Disease-10 (ICD-10); HAMD-17≥17; HCs with HAMD-17<7 and no history of any psychotic disorders; - Aged between 18 and 60 years (no limitation on gender); - Administration of SSRIs at least 6 weeks; - Participates undergoing MRI should be right-handed and free of traumatic brain injury, claustrophobia or metal implants; - Written informed consents are obtained by participates. | - **ICD-10 diagnoses: schizophrenia, bipolar disorder, manic episode or other psychotic disorders; alcohol and drug addiction; a current substance use disorder and lifetime history of substance abuse;** - **Significant skin lesions, severe allergic diseases, tumors, and severe or unstable internal diseases involving the cardiovascular, digestive, endocrine, or hematological system;** - **Positive suicidal tendency;** - **Allergy to adhesive tape, fear of intradermal acupuncture;** - **Pregnancy and lactation;** - **Mental retardation and difficulty cooperating with doctors;** - **Previously treated with intradermal acupuncture or participating in other clinical trials.** |
| MDD, major depressive disorder; HCs, health controls; SSRIs, selective serotonin reuptake inhibitors; | |

****eTable 2. Acupoints Locations****

| **Acupoints** | **Code** | **Localization** |
| --- | --- | --- |
| Shenmen | HT7 | Located on the palmar ulnar end of the transverse crease of the wrist and on the radial aspect of the tendon of the ulnar flexor. |
| Neiguan | PC6 | Located 2 cun above the transverse wrist crease between the tendons of the palmaris longus and flexor carpi radialis. |
| Sanyinjiao | SP6 | Located 3 cun directly above the tip of the medial malleolus, on the posterior border of the tibia. |
| Taichong | LR3 | Located on the dorsal side of the foot and sunken behind the first metatarsal space. |

****eTable 3. Coordinates for ROI****

| **ROI** | **Coordinates (mm)** | | |
| --- | --- | --- | --- |
|  | **X** | **Y** | **Z** |
| Striatum |  |  |  |
| VSi | ±9 | 9 | -8 |
| VSs | ±10 | 15 | 0 |
| DC | ±13 | 15 | 9 |
| DCP | ±28 | 1 | 3 |
| DRP | ±25 | 8 | 6 |
| VRP | ±20 | 12 | -3 |
| VTA | ±12 | -18 | -20 |
| DRN | 0 | -27 | -9 |
| MRN | 0 | -31 | -21 |
| ROI, regions of interest; VSi, ventral caudate/nucleus accumbens (inferior); VSs, ventral caudate (superior); DC, dorsal caudate; DCP, dorsal caudal putamen; DRP, dorsal rostral putamen;VRP, ventral rostral putamen; VTA, ventral tegmental area; DRN, dorsal raphe nucleus; MRN, median raphe nucleus. | | | |

****eTable 4. Comorbid conditions at baseline****

| ****Comorbid conditions**** | **SSRIs**  **(n=40)** | **SSRIs+SIA (n=40)** | **SSRIs+AIA (n=40)** | **Total (n=120)** |
| --- | --- | --- | --- | --- |
| Anxiety disorder, no. (%) | 30 (75.0) | 27 (67.5) | 25 (62.5) | 82 (68.3) |
| Sleep disorder, no. (%) | 24 (60.0) | 22 (55.0) | 23 (57.5) | 69 (57.5) |
| Cognitive impairment, no. (%) | 7 (17.5) | 6 (15.0) | 4 (10.0) | 17 (14.2) |
| Somatization disorder, no. (%) | 11 (27.5) | 12 (30.0) | 12 (30.0) | 35 (29.2) |
| Eating disorder, no. (%) | 3 (7.5) | 4 (10.0) | 3 (7.5) | 10 (8.3) |
| SSRIs, selective serotonin reuptake inhibitors; SIA, sham intradermal acupuncture; AIA, active intradermal acupuncture. | | | | |
|  | | | | |

****eTable 5. Acupuncture expectations at baseline****

| **Do you think acupuncture will be effective in improving MDD symptoms?** | **SSRIs (n=40)** | **SSRIs+SIA (n=40)** | **SSRIs+AIA (n=40)** | **Total (n=120)** |
| --- | --- | --- | --- | --- |
| Very effective (score 4), no. (%) | 12 (30.0) | 11 (27.5) | 15 (37.5) | 38 (31.7) |
| Possibly effective (score 3), no. (%) | 15 (37.5) | 13 (32.5) | 17 (42.5) | 45 (37.5) |
| Inconclusive (score 2), no. (%) | 12 (30.0) | 15 (37.5) | 6 (15.0) | 33 (27.5) |
| Possibly ineffective (score 1), no. (%) | 0 (0.0) | 1 (2.5) | 1 (2.5) | 2 (1.7) |
| Ineffective (score 0), no. (%) | 1 (2.5) | 0 (0.0) | 1 (2.5) | 2 (1.7) |
| MDD, major depressive disorder; SSRIs, selective serotonin reuptake inhibitors; SIA, sham intradermal acupuncture; AIA, active intradermal acupuncture. | | | | |
|  | | | | |

****eTable 6. Baseline characteristics in the MRI study****

| **Characteristics** | **HC（n=20）** | **MDD（n=20）** | ***P*** |
| --- | --- | --- | --- |
| Age (years), mean (SD) | 25.6 (3.7) | 25.1 (5.4) | 0.734^a^ |
| Sex, no. (%) |  |  | 1.000^b^ |
| Female | 7 (35.0) | 7 (35.0) |  |
| Male | 13 (65.0) | 13 (65.0) |  |
| BMI (kg/m^3^), mean (SD) | 20.4 (1.8) | 20.4 (2.7) | 0.986^a^ |
| HAMD-17 score, mean (SD) | 23.2 (4.0) | 1.9 (1.5) | 0.000^a^ |
| HC, health control; MDD, major depressive disorder; BMI, body mass index; HAMD-17, Hamilton Depression Rating Scale-17.  ^a^Evaluated by the Independent Samples *T*-test; ^b^Evaluated by the Pearson's Chi-squared test. | | | |

****eTable 7. Change in HAMD-17 five factor scores over time****

| **Variables** | **SSRIs**  **(n=36)** | **SSRIs+SIA (n=37)** | **SSRIs+AIA (n=36)** | **Inter-group differences^a^** | | **Repeatedly measured data^b^** | | **Between-group comparisons** | | | |
| --- | --- | --- | --- | --- | --- | --- | --- | --- | --- | --- | --- |
|  |  |  |  | ***F*** | ***P*** | ***F*** | ***P*** | **SSRIs+AIA vs. SSRIs** | | **SSRIs+AIA vs. SSRIs+SIA** | |
|  |  |  |  |  |  |  |  | **MD (95% CI)^c^** | **Effect size^d^** | **MD (95% CI)^c^** | **Effect size^d^** |
| Anxiety/  Somatization |  |  |  |  |  | 4.83 | ＜0.001 |  |  |  |  |
| Week 3 | -1.0 (0.3)^†^ | -1.1 (0.3)^†^ | -1.8 (0.3)^†^ | 1.64 | 0.199 |  |  | -0.8 (-1.9 to -0.4) | -0.38 | -0.7 (-1.8 to -0.5) | -0.34 |
| Week 6 | -1.4 (0.4)^†^ | -1.2 (0.4)^†^ | -3.5 (0.4)^†*#^ | 9.36 | ＜0.001 |  |  | -2.1 (-3.5 to -0.7) | -0.84 | -2.2 (-3.6 to -0.8) | -0.89 |
| Week 10 | -2.3 (0.4)^†^ | -1.9 (0.4)^†^ | -3.4 (0.4)^†#^ | 4.03 | 0.021 |  |  | -1.1 (-2.5 to 0.3) | -0.48 | -1.6 (-3.0 to -0.2) | -0.60 |
| Weight |  |  |  |  |  | 1.63 | 0.151 |  |  |  |  |
| Week 3 | -0.3 (0.1) | 0.0 (0.1) | -0.2 (0.1) | 1.73 | 0.183 |  |  | 0.1 (-0.3 to 0.5) | 0.11 | -0.2 (-0.6 to 0.2) | -0.34 |
| Week 6 | -0.2 (0.1) | -0.1 (0.1) | -0.4 (0.1)^†^ | 1.77 | 0.176 |  |  | -0.2 (-0.6 to 0.2) | -0.25 | -0.3 (-0.7 to -0.1) | -0.50 |
| Week 10 | -0.3 (0.1) | -0.2 (0.1) | -0.5 (0.1)^†^ | 1.75 | 0.178 |  |  | -0.2 (-0.6 to 0.2) | -0.24 | -0.3 (-0.7 to -0.1) | -0.51 |
| Cognitive impairment |  |  |  |  |  | 1.86 | 0.115 |  |  |  |  |
| Week 3 | -0.8 (0.2)^†^ | -0.5 (0.2)^†^ | -1.1 (0.2)^†^ | 1.76 | 0.177 |  |  | -0.3 (-1.0 to 0.4) | -0.20 | -0.5 (-1.2 to 0.2) | -0.43 |
| Week 6 | -0.8 (0.3)^†^ | -1.1 (0.3)^†^ | -1.7 (0.3)^†^ | 3.02 | 0.053 |  |  | -0.9 (-1.8 to 0.0) | -0.52 | -0.6 (-1.5 to 0.3) | -0.41 |
| Week 10 | -1.6 (0.3)^†^ | -1.3 (0.3)^†^ | -1.9 (0.3)^†^ | 1.20 | 0.305 |  |  | -0.3 (-1.3 to 0.7) | -0.16 | -0.6 (-1.7 to 0.4) | -0.37 |
| Retardation |  |  |  |  |  | 2.95 | 0.016 |  |  |  |  |
| Week 3 | -0.9 (0.2)^†^ | -0.8 (0.2)^†^ | -1.6 (0.2)^†^ | 3.26 | 0.042 |  |  | -0.6 (-1.4 to 0.1) | -0.47 | -0.7 (-1.5 to 0.0) | -0.55 |
| Week 6 | -1.4 (0.3)^†^ | -1.5 (0.3)^†^ | -2.7 (0.3)^†*#^ | 7.35 | 0.001 |  |  | -1.3 (-2.1 to -0.4) | -0.82 | -1.2 (-2.0 to -0.3) | -0.76 |
| Week 10 | -2.1 (0.3)^†^ | -1.9 (0.3)^†^ | -3.1 (0.3)^†#^ | 4.04 | 0.020 |  |  | -1.0 (-2.1 to 0.1) | -0.60 | -1.0 (-2.2 to -0.1) | -0.59 |
| Sleep disturbance |  |  |  |  |  | 0.85 | 0.519 |  |  |  |  |
| Week 3 | -0.3 (0.2) | -0.4 (0.2) | -0.6 (0.2) | 0.43 | 0.651 |  |  | -0.3 (-1.1 to 0.5) | -0.20 | -0.2 (-1.0 to 0.5) | -0.17 |
| Week 6 | -0.8 (0.3)^†^ | -0.9 (0.3)^†^ | -1.4 (0.3)^†^ | 1.74 | 0.181 |  |  | -0.7 (-1.6 to 0.3) | -0.38 | -0.6 (-1.5 to 0.4) | -0.37 |
| Week 10 | -1.0 (0.3)^†^ | -1.2 (0.3)^†^ | -1.6 (0.3)^†^ | 0.99 | 0.374 |  |  | -0.6 (-1.7 to 0.5) | -0.32 | -0.4 (-1.4 to 0.7) | -0.21 |
| Abbreviations: SSRIs, selective serotonin reuptake inhibitors; SIA, sham intradermal acupuncture; AIA, active intradermal acupuncture.  ^*^*P*＜0.05 vs. SSRIs group, ^#^*P*＜0.05 vs. SSRIs+SIA group; ^†^*P*＜0.05 for intra-group comparisons.  ^a^Inter-group differences were evaluated by the one factorial *ANOVA* test; ^b^Repeatedly measured data (10 weeks’ period) were assessed by the two-way repeated measures *ANOVA* test; ^c^Between-group comparisons were adjusted by Bonferroni test; ^d^Effct size was calculated using *Cohen’s d*. | | | | | | | | | | | |

****eTable 8. Comorbid medications taken by patients****

| ****Medications**** | ****SSRIs (n=40)**** | ****SSRIs+SIA (n=40)**** | ****SSRIs+AIA (n=40)**** | ****Total (n=120)**** |
| --- | --- | --- | --- | --- |
|  |  |  |  |  |
| Use of sedative-hypnotics, no.(%) | 16 (40.0) | 19 (47.5) | 11 (27.5) | 46 (38.3) |
| Alprazolam | 5 (12.5) | 6 (15.0) | 3 (7.5) | 14 (11.6) |
| Oxazepam | 4 (10.0) | 4 (10.0) | 4 (10.0) | 12 (10.0) |
| Lorazepam | 3 (7.5) | 4 (10.0) | 1 (2.5) | 8 (6.7) |
| Zopiclone | 3 (7.5) | 3 (7.5) | 2 (5.0) | 8 (6.7) |
| Zolpidem | 1 (2.5) | 2 (5.0) | 1 (2.5) | 4 (3.3) |
| Use of antipsychotics, no.(%) | 9 (22.5) | 7 (17.5) | 8 (20.0) | 24 (20.0) |
| Aripiprazole | 0 (0.0) | 1 (2.5) | 2 (5.0) | 3 (2.5) |
| Olanzapine | 2 (5.0) | 2 (5.0) | 2 (5.0) | 6 (5.0) |
| Quetiapine | 7 (17.5) | 4 (10.0) | 4 (10.0) | 15 (12.5) |
| SSRIs, selective serotonin reuptake inhibitors; SIA, sham intradermal acupuncture; AIA, active intradermal acupuncture. | | | | |

****eTable 9. Other clinical outcomes****

| **Outcomes** | **SSRIs**  **(n=40)** | **SSRIs+SIA (n=40)** | **SSRIs+AIA (n=40)** | **Between-group comparisons, OR (95% CI)** | |
| --- | --- | --- | --- | --- | --- |
|  |  |  |  | **SSRIs+AIA**  **vs. SSRIs** | **SSRIs+AIA**  **vs. SSRIs+SIA** |
| IA compliance (%), mean (SD) | — | 92.8 (18.5) | 92 (20.7) | — | 4.4 (-8.0 to 9.5) |
| IA blind success, no.(%) | — | 36 (90.0) | 37 (92.5) | — | 0.7 (0.2 to 3.5) |
| SSRIs, selective serotonin reuptake inhibitors; SIA, sham intradermal acupuncture; AIA, active intradermal acupuncture; OR, odds ratio; MD, mean difference. | | | | | |

****eTable 10. FC differences between MDD and HC****

| **Comparisons** | **ROI** | **Brain regions** | **MNI coordinates** | | | **Cluster size** | ***Z*** |
| --- | --- | --- | --- | --- | --- | --- | --- |
|  |  |  | **X** | **Y** | **Z** |  |  |
| MDD *>* HC | VSs_L | ParaHippocampal_L | -18 | -24 | -3 | 371 | 4.62 |
|  |  | Paracentral_Lobule_R | 9 | -24 | 72 | 990 | 4.06 |
|  | VSs_R | Postcentral_L | -39 | -24 | 42 | 599 | 4.55 |
|  |  | Paracentral_Lobule_R | 6 | -30 | 69 | 367 | 4.13 |
|  | VSi_R | Frontal_Inf_Tri_L | -45 | 36 | 15 | 584 | 4.05 |
|  | DC_L | Cerebelum_6_R | 30 | -78 | -21 | 408 | 4.35 |
|  |  | Lingual_L | -15 | -96 | -12 | 382 | 4.69 |
|  | DC_R | Frontal_Mid_L | -45 | 18 | 39 | 486 | 4.19 |
|  | DRP_R | Frontal_Inf_Tri_L | -48 | 24 | 18 | 465 | 4.07 |
|  | VRP_R | Frontal_Mid_R | 36 | 21 | 39 | 757 | 4.36 |
|  |  | Frontal_Inf_Tri_L | -48 | 21 | 21 | 480 | 6.04 |
|  | VTA_L | Precuneus_L | 0 | -57 | 21 | 487 | 3.43 |
|  | VTA_R | Postcentral_L | -21 | -39 | 54 | 948 | 5.11 |
|  | DRN | Precentral _L | -21 | -12 | 75 | 607 | 3.76 |
|  | MRN | Frontal_Inf_Orb_R | 30 | 27 | -21 | 573 | 4.82 |
| MDD *<* HC | VSs_L | Frontal_Mid_Orb_R | 33 | 54 | -9 | 442 | -4.57 |
|  | VSs_R | Frontal_Inf_Orb_L | -36 | 24 | -9 | 901 | -6.37 |
|  |  | Frontal_Mid_Orb_R | 33 | 54 | -6 | 826 | -5.42 |
|  | VSi_L | Frontal_Sup_Orb_L | -21 | 60 | -3 | 406 | -4.04 |
|  | VSi_R | Frontal_Sup_L | -21 | 48 | 45 | 883 | -5.09 |
|  |  | Frontal_Sup_Orb_L | -24 | 12 | -15 | 424 | -5.35 |
|  | DC_L | Precuneus_L | -3 | -57 | 54 | 922 | -4.38 |
|  | DC_R | Insula_R | 30 | 12 | -15 | 521 | -5.20 |
|  |  | Occipital_Mid_L | -36 | -78 | 15 | 438 | -4.52 |
|  |  | Precuneus_L | -12 | -54 | 48 | 565 | -4.13 |
|  | DCP_R | Occipital_Mid_R | 30 | -93 | 18 | 592 | -4.80 |
|  | DRP_R | Frontal_Sup_Medial_L | -9 | 66 | 21 | 478 | -4.04 |
|  | VRP_L | Rectus_R | 3 | 48 | -18 | 549 | -3.96 |
|  | VRP_R | Frontal_Sup_Medial_L | -6 | 66 | 18 | 577 | -4.55 |
|  | MRN | Cerebelum_4_5_R | 9 | -48 | -24 | 510 | -3.72 |
| ROI, regions of interest; FC, functional connectivity; HC, health control; MDD, major depressive disorder; VSi, ventral caudate/nucleus accumbens (inferior); VSs, ventral caudate (superior); DC, dorsal caudate; DCP, dorsal caudal putamen; DRP, dorsal rostral putamen;VRP, ventral rostral putamen; VTA, ventral tegmental area; DRN, dorsal raphe nucleus; MRN, median raphe nucleus; L, left; R, right. | | | | | | | |
|  | | | | | | | |

****eTable 11. FC difference between pre- and post-IA intervention****

| **Comparisons** | **ROI** | **Brain regions** | **MNI coordinates** | | | **Cluster size** | ***Z*** |
| --- | --- | --- | --- | --- | --- | --- | --- |
|  |  |  | **X** | **Y** | **Z** |  |  |
| post-IA *>* pre-IA | DRN | Calcarine_R | 24 | -51 | 9 | 518 | 4.05 |
| post-IA *<* pre-IA | VSi_L | Cerebelum_9_L | -9 | -36 | -48 | 705 | -5.84 |
|  | VRP_R | Frontal_Inf_Tri_R | 45 | 27 | 9 | 698 | -5.18 |
|  | VTA_R | Caudate_R | 3 | 9 | 0 | 978 | -5.71 |
|  | MRN | Frontal_Sup_Orb_L | -18 | 60 | -9 | 1981 | -5.02 |
| ROI, regions of interest; FC, functional connectivity; DRN, dorsal raphe nucleus; VSi, ventral caudate/nucleus accumbens (inferior); VRP, ventral rostral putamen; VTA, ventral tegmental area; MRN, median raphe nucleus; L, left; R, right; | | | | | | | |
